# Regulatory Roles of Antimicrobial Peptides in the Nervous System: Implications for Neuronal Aging

**DOI:** 10.3389/fncel.2022.843790

**Published:** 2022-03-07

**Authors:** Bradey A. R. Stuart, Ariel L. Franitza, Lezi E

**Affiliations:** ^1^Neuroscience Research Center, Medical College of Wisconsin, Milwaukee, WI, United States; ^2^Department of Cell Biology, Neurobiology and Anatomy, Medical College of Wisconsin, Milwaukee, WI, United States

**Keywords:** antimicrobial peptide (AMP), aging, neurodegeneration, neurodegenerative diseases, Alzheimer's disease, nervous system, neuropeptide

## Abstract

Antimicrobial peptides (AMPs) are classically known as important effector molecules in innate immunity across all multicellular organisms. However, emerging evidence begins to suggest multifunctional properties of AMPs beyond their antimicrobial activity, surprisingly including their roles in regulating neuronal function, such as sleep and memory formation. Aging, which is fundamental to neurodegeneration in both physiological and disease conditions, interestingly affects the expression pattern of many AMPs in an infection-independent manner. While it remains unclear whether these are coincidental events, or a mechanistic relationship exists, previous studies have suggested a close link between AMPs and a few key proteins involved in neurodegenerative diseases. This review discusses recent literature and advances in understanding the crosstalk between AMPs and the nervous system at both molecular and functional levels, with the aim to explore how AMPs may relate to neuronal vulnerability in aging.

## 1. Introduction

Antimicrobial peptides (AMPs) are short, typically cationic peptides that are found in every kingdom of life and were originally discovered as host defense peptides. As part of the innate immune system, AMPs fight off pathogens such as bacteria, fungi, parasites, and enveloped viruses, through their insertion and subsequent disruption of the membrane structure of microbe, or having interactions with microbial intracellular/intraviral components, or a combination of both (Raheem and Straus, 2019; Benfield and Henriques, 2020). Their ability to directly act upon and kill pathogens makes them an ideal target for use as therapeutic anti-infectives, and a potential alternative to traditional antibiotics, resulting in numerous rational design studies to create synthetic AMPs (Mahlapuu et al., 2016, 2020; Cardoso et al., 2020). With over 3000 characterized AMPs to date (Antimicrobial Peptide Database, 2021, https://aps.unmc.edu/), AMPs are typically classified according to their structure and source, but other classifications include their amino acid composition or activity against different types of pathogens (Huan et al., 2020). For example, in human, some of the most prevalent and well-studied AMPs are the defensins, and the cathelicidin LL-37, which have a beta-sheet or alpha-helix motif, respectively (Wang, 2014). LL-37 is expressed ubiquitously in tissues throughout the body including epithelial cells, immune cells, and the nervous system (Dürr et al., 2006; Lee et al., 2015), while other AMPs have the greatest abundance in barrier tissues where exposure to microbes is frequent, such as the skin, airways, and intestinal epithelium (Laube et al., 2006; Kenshi and Richard, 2008; Muniz et al., 2012). For an in-depth discussion of the mechanisms of actions in the immune system and classification of various AMPs, which is out of the scope of current discussion, see the following reviews (Huan et al., 2020; Zhang et al., 2021).

AMPs have immense functional diversity. In addition to their antimicrobial function, a number of AMPs can act as anti-cancer, anti-biofilm, anti-diabetic, and wound healing agents (Mangoni et al., 2016; Di Somma et al., 2020; Tornesello et al., 2020; Soltaninejad et al., 2021). Surprisingly, recent work begins to suggest a potential alternative role of AMPs as signaling molecules to regulate cell behaviors in various tissues. In this review, we highlight and focus on recent advances in understanding the alternative roles of AMPs in the nervous system, particularly within the context of aging and neurodegenerative diseases, and summarize the open questions of this rapidly expanding field.

## 2. AMPs and Nervous System

Although classically thought of as the first line of defense between the environment and our own barrier tissues, emerging evidence suggests an intricate link between AMPs and the nervous system. For example, psychological stress dramatically downregulates the expression of cathelin-related AMP (CRAMP) in the skin of mice, increasing their susceptibility to cutaneous infections, and transforming growth factor-beta signaling from neurons non-cell autonomously regulates the AMP *cnc-2* expression in the skin of *Caenorhabditis elegans (C. elegans)* (Aberg et al., 2007; Zugasti and Ewbank, 2009) (Table 1). Additionally, work by E et al. (2018) and Sinner et al. (2021) reveals that a skin-expressed AMP, neuropeptide-like protein 29 (NLP-29), has specific functions in inducing neurodegeneration and promoting sleep in *C. elegans*. Together, these data suggest the involvement of AMPs in the bidirectional interactions between the nervous system and non-neuronal tissues. In this section we explore the cellular and molecular mechanisms underlying such interactions, by specifically discussing the roles of AMPs in modulating neuroinflammation, their similarity to neuropeptides, and their functions in the central nervous system (CNS).

**Table 1:** Selected AMPs discussed in this review.

**AMP and AMP- like protein**	**Host organism**	**Structural motif**	**Charge**	**Tissue expressed in**	**Functions**	**Orthologues**
LL-37	Human	Alpha helix	+6	Lung, colon, esophagus, skin, eyes, CNS, immune cells, epithelia (Dürr et al., 2006; Lee et al., 2015)	Host defense, nucleic acid presentation, immunomodulation, chemotaxis (Kahlenberg and Kaplan, 2013)	Rat, mouse, chicken, rabbit, dog, vertebrates (Scheenstra et al., 2020)
hBD-1,2,3	Human	Alpha helix and beta sheets	+4-11	Skin, lung, trachea, eyes, colon, CNS, testis, immune cells (Pazgier et al., 2006)	Host defense, wound healing, chemokine, angiogenesis (Ghosh et al., 2019)	Plants, vertebrates, invertebrates, insects (Pazgier et al., 2006)
PACAP	Human	Alpha helix	+11	Nervous system (Hirabayashi et al., 2018)	Neuropeptide, proliferation, metabolism, apoptosis, differentiation, immune system, potential host defense (Sherwood et al., 2000; Lee et al., 2021)	Vertebrates, invertebrates, *Drosophila*, chicken, lizard, frog, fish (Montero et al., 2000)
NLP-29	*C. elegans*	Predicted alpha helix	+6	Skin (Pujol et al., 2008)	Host defense, wound healing, sleep, neuronal aging (Pujol et al., 2008; E et al., 2018; Sinner et al., 2021)	Nematode
CNC-2	*C. elegans*	Predicted alpha helix	+3	Skin (Zugasti and Ewbank, 2009)	Host defense against fungal infection (Zehrbach et al., 2017)	Nematode
Metchnikowin	*Drosophila*	Alpha helix	+2	Fat body, epithelia	Host defense, mortality following TBI, potentially cytotoxic, potentially involved in sleep (Dissel et al., 2015; Badinloo et al., 2018; Swanson et al., 2020)	Insect (Buonocore et al., 2021)
Nemuri	*Drosophila*	Predicted alpha helix	+14	N.D. Accumulates in CNS	Host defense, sleep (Toda et al., 2019)	Insect, sequence similarity to fish cathelicidins (Toda et al., 2019)
Drosocin	*Drosophila*	Predicted alpha helix	+5	Fat body, epithelia	Host defense, lifespan, potentially involed in sleep (Lazzaro and Clark, 2003; Dissel et al., 2015; Loch et al., 2017)	Insect (Buonocore et al., 2021)
AttacinA	*Drosophila*	Predicted alpha helix	+3	Fat body, epithelia	Host defense, lifespan (Badinloo et al., 2018; Buonocore et al., 2021)	Insect (Buonocore et al., 2021)
Cecropin A1	*Drosophila*	Predicted alpha helix	+8	Fat body, epithelia	Host defense, lifespan, potential cytotoxicity (Badinloo et al., 2018)	Insect (Buonocore et al., 2021)
Diptericin B	*Drosophila*	Unknown	+7	Head fat body	Host defense, memory formation (Barajas-Azpeleta et al., 2018)	Insect (Buonocore et al., 2021)
GNBP-like3	*Drosophila*	Unknown	+5	Nervous system	Host defense, memory formation (Barajas-Azpeleta et al., 2018)	Insect
NDA-1	*Hydra Vulgaris*	Predicted beta sheet	N.D	Nervous system	Neuropeptide, modulates microbiome (Augustin et al., 2017)	N/A *Hydra* Specific

*CNC-2, caenacin-2; CNS, central nervous system; GNBP, Gram-negative bacteria-binding protein; hBD, human beta-defensin; N.D., not determined; N/A, not available; NLP-29, neuropeptide-like protein 29; PACAP, pituitary adenylate-cyclase-activating polypeptide*.

### 2.1. AMPs and Neuroinflammation

Inflammation of the brain and spinal cord is referred to as neuroinflammation, and much of this response is generated through the action of the CNS resident macrophages, known as microglia. Neuroinflammation can be induced in response to infection, injury, stress, and aging (DiSabato et al., 2016). Although it is important in recovery and protection of the nervous system, uncontrolled, excessive neuroinflammation can lead to cellular damage (Cherry et al., 2014; Kielian, 2014) and is implicated in a broad spectrum of neurological disorders, from traumatic to chronic neurodegenerative and ischemic brain damage.

Pattern recognition receptors (PRRs) are important mediators of inflammation that respond to exogenous infectious ligands (pathogen-associated molecular patterns, PAMPs), such as lipopolysaccharide (LPS), and endogenous molecules that are released during tissue/cellular damage (damage-associated molecular patterns, DAMPs) (Amarante-Mendes et al., 2018). Toll-like receptors (TLRs), a family of transmembrane proteins that respond to a variety of PAMPs and DAMPs, are one of the most well studied PRRs (Hanke and Kielian, 2011; Kawasaki and Kawai, 2014). When activated, TLRs, which are expressed in both non-immune cells and immune cells including microglia, can lead to an increase in a series of inflammatory signaling molecules (Kawasaki and Kawai, 2014) as well as AMPs in human and mice (Thoma-Uszynski et al., 2001; Hertz et al., 2003; Rivas-Santiago et al., 2008).

Clinically, the levels of the AMPs LL-37 and defensins increase in the cerebrospinal fluid (CSF) in response to a bacterial meningitis challenge, and the CSF of patients with bacterial meningitis has antimicrobial activities against both Gram-positive and -negative bacteria (Maffei et al., 1999; Brandenburg et al., 2008), suggesting AMPs may directly contribute to the killing of pathogens in the nervous system. Interestingly, however, AMPs can be both anti-inflammatory and pro-inflammatory in the nervous system, depending on the context. In support of the anti-inflammatory properties of AMPs, intracerebroventricular infusion of CRAMP decreases the mortality rate in a mouse model of bacterial meningitis, likely associated with the reduction in the abundance of pro-inflammatory cytokines, tumor necrosis factor-α (TNF-α) and interleukin-6 (IL-6), specifically in the hippocampus (Dörr et al., 2015). Ligand binding and activation of TLR2 and TLR4 typically causes an increase in pro-inflammatory cytokines (Akira and Takeda, 2004; Kawasaki and Kawai, 2014). Cationic AMPs have been shown to bind and sequester free bacterial LPS and lipoteichoic acid (LTAs) to prevent the activation of TLR2 and TLR4 (Scott et al., 1999; Sun and Shang, 2015), possibly explaining by what mechanism AMPs may mediate an anti-inflammatory response in the nervous system. Paradoxically, the CRAMP treatment in the meningitis model increases the mRNA levels of TLR2 and TLR4 (Dörr et al., 2015). On one hand, this could be anti-inflammatory, as high expression of TLRs can serve as soluble decoys to bind excess PAMP ligands (Iwami et al., 2000; Lai and Gallo, 2008), which would decrease pro-inflammatory signaling. Alternatively, this could also suggest a pro-inflammatory role of AMPs, as higher levels of TLR2 and TLR4 may lead to an increased sensitivity to bacterial challenge, enhancing pro-inflammatory signaling. In support of these pro-inflammatory properties, LL-37 may be secreted from human neuronal cells upon stress or damage, to stimulate glial cells to release pro-inflammatory cytokines and chemokines, which in turn decreases neuronal viability *in vitro* (Lee et al., 2015). This raises the possibility that LL-37 acts as a signaling molecule to activate glial cells within the CNS.

Although it is unknown which receptor(s) mediates LL-37-induced glial activation, in other cell types LL-37 has been shown to activate a variety of G protein-coupled receptors (GPCRs), receptor tyrosine kinases, ligand-gated ion channels, or TLRs (Larrick et al., 1991; Elssner et al., 2004; Brandenburg et al., 2010; Verjans et al., 2016) which may be potential targets. Previous evidence shows that LL-37 can function as chemoattractant to neutrophils and eosinophils in other tissues through its interactions with formyl peptide receptors, a class of GPCRs involved in chemotaxis (Tjabringa et al., 2006; Hemshekhar et al., 2018) which are expressed in microglia as well (Iribarren et al., 2005). It is an open question of whether expression of LL-37 also maintains its chemoattractant properties in its neuroinflammatory context. Curiously, LL-37 and other AMPs can form large crystalline complexes with endogenous or foreign dsDNA, dsRNA, ssDNA, and ssRNA, which then amplify inflammation through their interactions with TLR3, TLR9, and TLR7/8 [see (Lee et al., 2019) for a comprehensive review]. This surprising role in interacting with nucleic acids, and subsequent presentation to TLRs that mediate pro-inflammatory responses, further complicates the mechanisms of action of AMPs in neuroinflammation. The apparent diversity in whether AMPs act as “pro” or “anti” inflammatory molecules implies that AMPs may play a critical role in maintaining the immune homeostasis within the nervous system, and also emphasizes the importance of context and experimental setup when interpreting previous literature.

### 2.2. AMPs and Neuropeptides

Neuropeptides are an evolutionarily ancient and diverse set of messengers released from the nervous system that are critical in cell-to-cell signaling. After released from neurons, the majority of neuropeptides act upon one or more GPCRs on the surface of the target cell to initiate downstream signaling (Russo, 2017). Neuropeptides are present both in the CNS and peripheral nervous system (PNS) and have various functions in regulating emotion, pain, digestion, and behavior (Holzer and Farzi, 2014; Kash et al., 2015; Russo, 2017). Intriguingly, neuropeptides and AMPs have remarkable structural similarities, including amphipathicity, net cationic charge, amino acid composition, and size (Brogden et al., 2005). Furthermore, multiple neuropeptides, including but not limited to human Neuropeptide Y (NPY), substance P, and α-Melanocyte stimulating hormone, demonstrate antimicrobial activity in *in vitro* assays (Kowalska et al., 2002; Hansen et al., 2006; Lee and Herzog, 2009; Shireen et al., 2009). In mammals, one particularly noteworthy neuropeptide with antimicrobial properties is pituitary adenylate cyclase-activating polypeptide (PACAP) (Table 1). It has been well established that PACAP promotes differentiation of neural progenitor cells (Hirose et al., 2006), cell survival, and neurite growth (Gonzalez et al., 1997; Shioda et al., 2006; Kaneko et al., 2018). However, a recent study shows that, upon infection by *Staphylococcus aureus* or *Candida albicans*, PACAP is specifically induced in mouse brain, implying a multi-functional purpose of these neuropeptides in the intersections of immune and nervous systems (Shioda et al., 2006; Kaneko et al., 2018; Lee et al., 2021). Although it currently remains unknown whether many of these neuropeptides' *in vitro* antimicrobial activity translates to *in vivo* function, at least one neuropeptide with antimicrobial properties, NDA-1, from the model organism *Hydra vulgaris* contributes to the killing of Gram-positive bacteria to specifically shape a balanced microbiome on their body surface (Augustin et al., 2017) (Table 1). This shared function between AMPs and neuropeptides suggests possible evolutionary conservation of their roles and mechanisms of action in the innate immune system. Much like AMPs, neuropeptides also have immunomodulatory roles (Souza-Moreira et al., 2011; Chen et al., 2020). Considering approximately 100 neuropeptides out of the 1,000+ small peptides predicted in the human genome have been studied (Russo, 2017), it is likely we will identify more neuropeptides with antimicrobial function in the future, potentially blurring the lines of how these peptides are defined and classified. Thus, it is tempting to speculate that AMPs may serve as the non-neuronal tissue analogues to neuropeptides to act as signaling molecules, mediators of the immune system, and neuromodulators.

### 2.3. AMPs and CNS Function

Although many AMPs are expressed where interactions with microbes is frequent, as discussed above, a growing experimental body of work suggests AMPs also exist in the CNS. Because of their lack of an adaptive immunity, the fruit fly *Drosophila melanogaster* has become a powerful tool for understanding the role of innate immunity components, especially AMPs in modulating the CNS (Imler and Bulet, 2005; Hanson and Lemaitre, 2020). Using this model organism, studies have discovered two AMPs functioning as necessary components of long-term memory formation. An mRNA sequencing-based screen has identified Diptericin B (DptB) as being significantly upregulated following different paradigms of behavioral training (Barajas-Azpeleta et al., 2018) (Table 1). Subsequent knockout of DptB demonstrates that it is required for long-term memory formation, while it is produced in the fat tissues of the head, suggesting that AMPs do not need to be produced by neural tissues to regulate CNS functions. This study also identifies Gram-negative bacteria-binding protein like 3 (GNBP-like3) as a neuronally expressed AMP that is also involved in modulating memory formation. Other examples of AMPs that have been shown to regulate CNS function in *Drosophila* include Nemuri, which induces sleep, and Metchnikowin, which appears to promote mortality following traumatic brain injury (Toda et al., 2019; Swanson et al., 2020) (Table 1). This evidence further highlights the possibility that AMPs may serve as essential signaling molecules in the CNS to regulate behaviors and maintain organismal homeostasis.

In mammals, it remains unclear whether these regulatory roles of AMPs in CNS functions are conserved, besides particitpating in neuroinflammation. Nevertheless, the expression of the human LL-37 homologue rCRAMP (rat CRAMP) has been detected within the CNS of murine models in an infection-independent manner (Maxwell et al., 2003; Bergman et al., 2005). Similarly in human, LL-37 has constitutive expression in the substantia nigra and sensory cortex (Lee et al., 2015), and human beta defensin-1 in the choroid plexus (Nakayama et al., 1999), despite the absence of apparent brain infections, brain injuries or other CNS disorders. What physiological role this expression in the mammalian CNS has, as of yet, remains undetermined, but we speculate that these or other AMPs are multifunctional and not limited to their microbicidal action. Behaviorally, in mice, psychological stress can reduce the expression of beta defensins in the skin, and depression can reduce alpha defensin expression in the intestine (Aberg et al., 2007; Suzuki et al., 2021). A postmortem study shows that human patients who died by suicide have decreased LL-37 expression in both dorsolateral prefrontal cortex and anterior cingulate cortex, which are critical for mood regulation (Bae et al., 2006), when compared to those who died by other causes (Postolache et al., 2020). Such evidence indicates an important correlation between AMPs and CNS functions in mammals, while it remains an area of investigation of whether they are parallel events or if there is a causal relationship. We posit that, considering other model organisms have AMPs whose actions in the nervous system originate from non-neuronal tissues (Barajas-Azpeleta et al., 2018; E et al., 2018), similar regulatory mechanisms may be conserved in mammals as well. Further work is necessary to uncover whether AMPs in human function in a physiological context to modulate CNS functions.

## 3. Implication of AMPs in Neurodegenerative Diseases

Neurodegeneration, the process of progressively losing the structure or function of neurons, is the key pathophysiological feature of neurodegenerative diseases such as Alzheimer's disease (AD), Huntington's disease, amyotrophic lateral sclerosis, and Parkinson's disease (PD). In each of these diseases, it is typical to see accumulation of proteins, such as amyloid beta (Aβ) or tau in AD, or alpha-synuclein in PD, in an altered conformation.

Although early hypotheses attempting to explain AD etiology implicate Aβ as the causative agent of the disease (Hardy and Higgins, 1992), multiple other hypotheses since propose other pathways, generating much debate (Du et al., 2018). More recently, a hypothesis known as the Antimicrobial Protection Hypothesis, which is extensively reviewed elsewhere (Moir et al., 2018), has garnered attention. Briefly, this hypothesis asserts that the expression and aggregation of Aβ is a response to pathogens, and Aβ oligomerization is protective against microbes, acting as an AMP. This hypothesis is based on the evidence that synthetic Aβ has antimicrobial properties against common pathogens *in vitro*, and that brain homogenates from AD patients have significantly higher antimicrobial activity than that from age-matched non-AD controls in an Aβ level-dependent manner (Soscia et al., 2010). Additionally, overexpression of Aβ in *C. elegans* protects them from *Candida albicans* infection, and overproduced Aβ in the transgenic 5xFAD mouse model promotes the animal survival against *Salmonella Typhimurium* infection, with the evidence showing colocalization of Aβ deposition and the invading bacteria (Kumar et al., 2016). Early cell culture studies also show that Aβ has the potential to bind and cause aggregates of viral particles, specifically influenza and herpes simplex virus-1 (HSV-1) (White et al., 2014; Bourgade et al., 2015, 2016). Also in 5xFAD mice, infection with HSV-1 promotes Aβ aggregation, and these aggregates appear to capture HSV-1 in *ex vivo* mouse brain tissue slices, as well as in human stem cell-derived neural cell culture (Eimer et al., 2018). However, recently repeated experiments with the same AD mouse model contradict these results. Bocharova et al. hypothesize that Aβ does not entrap viral particles, or protect against viral infection, and that such inconsistent observations may be explained by sex-specific expression of Aβ in 5xFAD mice or non-physiologically relevant levels of HSV-1 dosing used in the experiments (Bocharova et al., 2021). As such, the question of whether Aβ is protective, either in physiological or pathological contexts, remains vague. Elucidating the role of the antimicrobial properties of Aβ in human may lead to a more complete understanding of the factors causing AD and progression of AD and aid in the development of further therapeutic strategies.

Considering the aforementioned immunomodulatory roles of AMPs, both in the CNS and in other tissues, if Aβ was also a component of the innate immune system, one would expect Aβ to mediate inflammation in physiological conditions. Indeed, *in vitro* experiments show that microglia exposed to non-aggregated Aβ at low concentrations become reactive, and in turn express the AMP LL-37 as part of a pro-inflammatory response (Maezawa et al., 2011; Xu et al., 2018). Additionally, similar to human LL-37 being a chemoattractant to immune cells utilizing FPRL1 as a receptor (Yang et al., 2000), Aβ is a chemoattractant to mouse microglia by acting at the murine homologue of this GPCR (Tiffany et al., 2001). In human, Aβ's action upon the FPRL1 receptor causes internalization of Aβ into macrophages (Cui et al., 2002) and promotes inflammation (Schröder et al., 2020). Such interactions may provide context for the elusive role of the amyloid protein under physiological conditions. Much like the other AMPs discussed, Aβ can cause both pro or anti-inflammatory signaling depending on the context. On the anti-inflammatory side, *in vitro* studies demonstrate a direct interaction of LL-37 and Aβ, and that these interactions are protective, as LL-37 binding to Aβ decreases microglial-mediated toxicity against neuronal cells (De Lorenzi et al., 2017). Altogether, the current data suggests that Aβ may be an integral component of our innate immune system, and that there may be a balance between its interactions with other AMPs during the chronic neuroinflammation typical of AD and thus the progression of AD.

In other neurodegenerative diseases, the characteristic proteins also demonstrate antimicrobial action. In relation to PD, alpha-synuclein has *in vitro* antimicrobial activities against *Escherichia coli, Staphylococcus aureus*, and fungi (Park et al., 2016). In addition, the expression of alpha-synuclein in mice is also protective against RNA viruses from developing brain infections (Beatman et al., 2016). In a *Drosophila* model of the neurodegenerative disease ataxia-telangiectasia, ataxia-telangiectasia mutated (ATM) kinase results in increased AMP gene expression in glial cells, specifically through the NF-κB pathway (Petersen et al., 2012, 2013). Also, *Drosophila* carrying the mutant form of huntingtin, had impaired expression of a few AMPs, including DptB, Attacin, and cecropin A (Table 1), following bacterial infection (Lin et al., 2019), further suggesting a delicate interrelationship between the innate immune system and some of the key proteins in neurodegenerative diseases. The correlative and common elements between AMPs and neurodegenerative diseases warrants further investigation into whether AMPs are the drivers of the diseases, or misregulated as a consequence of the progression of diseases.

## 4. AMPs and Aging

### 4.1. AMP Expression in Development and Aging

**Figure 1:**
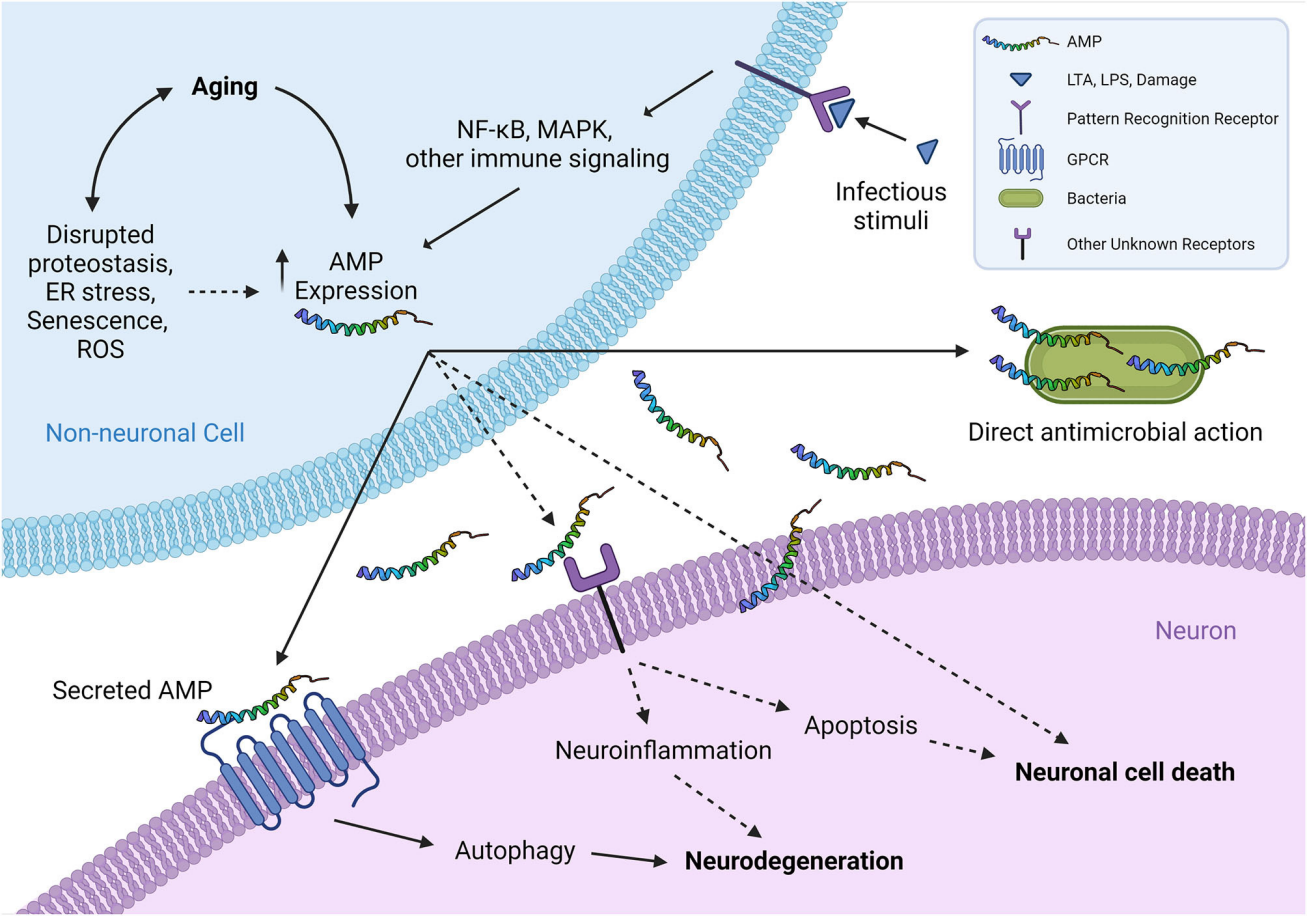
A schematic representation of proposed interplays between non-neuronal AMPs and neurons. The expression of AMPs from non-neuronal cells (such as epidermal and intestinal epithelial cells) typically increases in infections, which can be induced by exogenous infectious stimuli or endogenous molecules that are released subsequently by damaged cells binding to pattern recognition receptors (TLRs, etc.). Aging has recently been shown as another critical factor to induce the expression of multiple AMPs at both transcriptional and translational levels, across different species, in an infection-independent manner. Overproduced AMPs are secreted from non-neuronal cells and interact with neighboring neurons by binding and activating their specific neuronal cell surface receptors, including GPCRs, and possibly other receptors as well, such as receptor tyrosine kinases or ligand-gated ion channels. This ligand-receptor binding activates the downstream signaling that eventually leads to neurodegeneration-associated function decline and/or neuronal cell death. It is tempting to speculate that certain AMPs may affect neuronal health in a receptor-independent manner, for instance by disrupting neuron membranes in a similar fashion to their mechanism of action in killing pathogens. Although the cholesterol in higher eukaryotic cell membranes generally protects the cells from attacking by endogenous AMPs (Matsuzaki, 2009), age-related changes in biophysical properties of plasma membranes (Ledesma et al., 2012) presumably can increase the susceptibility to a direct toxic interaction with the overproduced AMPs in aging. (Illustration created with Biorender.com) Solid lines denote the mechanisms supported by experimental evidence from literature; dashed lines denote potential mechanisms proposed for future investigation to clarify. AMP, antimicrobial peptide; ER, endoplasmic reticulum; GPCR, G protein-coupled receptor; LTA, lipoteichoic acid; LPS, lipopolysaccharide; MAPK, mitogen-activated protein kinase; NF-κB, nuclear factor-B; ROS, reactive oxygen species; TLR, Toll-like receptor.

It has been the general consensus that AMPs are expressed at a relatively low, but constitutive level, especially in tissues that interact with the environment where microbial interactions are frequent. In the presence of infection, PAMPs on microbial surfaces typically activate specific receptors, such as TLRs, that lead to activation of transcription factors that increase the abundance of AMPs (Krisanaprakornkit et al., 2000; Rivas-Santiago et al., 2008; Gombart, 2009) (Figure 1). However, recent evidence has begun to implicate age as a factor in regulating the expression of AMPs as well. There appear to be two major increases in AMP expression that occur over the course of an organism's life. First, at the beginning of life, AMP expression seems to increase in the developing embryo or fetus, as evidenced in *Hydra*, mouse, chicken, and human (Gallo et al., 1997; Meade et al., 2009; Fraune et al., 2010; Gschwandtner et al., 2014). It is thought that the overexpression of AMPs “prepares” an embryo for its first encounter with microbes upon birth. However, considering the sterile environment of the chicken egg, the conserved nature of this expression, and the aforementioned alternative roles of AMPs in signaling, there may be a potential non-antimicrobial biological role of these peptides during embryogenesis. Consistent with this hypothesis, human fetal keratinocytes that are cultured in the presence of antibiotics still show strong AMP expression compared to that of neonatal or adult keratinocytes, suggesting an innate, young age-dependent expression mechanism (Gschwandtner et al., 2014). This developmental increase is followed by a marked decrease to a low or basal level of expression.

As organisms age, AMP expression again deviates from its baseline. Expression patterns of individual AMPs may differ, but in general, AMPs tend to gradually increase over the course of aging. One proposed mechanism of this apparent increase of AMP expression in aging populations is partially attributed to a compromised barrier between host and microbe, causing increased inflammation due to increased interactions with microbe-secreted signaling molecules. In support of this, many AMPs become dysregulated in aged mice, and these changes are associated with microbiome changes in the small intestine (Tremblay et al., 2017). In *Drosophila*, several studies show that aging specifically causes an increase in numerous AMPs (Kounatidis et al., 2017; Badinloo et al., 2018; Hanson and Lemaitre, 2020; Swanson et al., 2020; Wang et al., 2020). Surprisingly, flies grown in germ-free conditions still have age-dependent activation of AMP expression. Similarly, aging also causes a dramatic increase in AMP expression in other model organisms despite the absence of infection. Recently, we have found that the expression of the skin-expressed AMP NLP-29 gradually but significantly enhances over the course of aging in *C. elegans*, even with the treatment of antifungals and antibiotic compounds, suggesting the increase is specifically age dependent (E et al., 2018). Paradoxically, overexpression of certain AMPs (Attacin, Metchnikowin, cecropin A1, and Defensin) in *Drosophila* can impart cytotoxicity of muscle and fat cells, cognitive decline, and decreased lifespan (Kounatidis et al., 2017; Badinloo et al., 2018), while overexpression of other AMPs, such as Drosocin, extends *Drosophila* lifespan by specifically protecting the intestinal epithelium (Loch et al., 2017) (Table 1). These data suggest that individual AMPs may have context-specific and multifaceted physiological roles in aging.

To date, relatively few research studies have focused on elucidating the expression pattern of AMPs over the adult lifespan in human. It has been reported that the mRNA levels of human beta defensin 2 is elevated in peripheral blood mononuclear cells in aged populations (Castan̄eda-Delgado et al., 2013), while this observation may not be accurately reflected at the serum level (Castan̄eda-Delgado et al., 2017) (Table 1), as it is unclear whether any aging-associated defects complicate the peptide secretion process. However, it is noteworthy that, Aβ, which has recently been demonstrated as an AMP, gradually and significantly increases its level in multiple brain regions of human subjects over a healthy adult lifespan (Rodrigue et al., 2012). While these data suggest that the age-dependent expression pattern of AMPs observed in other organisms appear to be conserved in human to a certain extent, further research will be required to provide more comprehensive information on how AMPs are involved in human physiological aging at the functional level.

### 4.2. Contribution of AMP in Neuronal Aging

While we still face numerous challenges in dissecting the etiology and pathogenesis of neurodegenerative diseases, there is also a fundamental gap in our understanding on how functional aging of the nervous system is switched on. Emerging evidence indicates that the degeneration of neurons can be regulated by surrounding cells (microglia and astrocytes, etc.) at the cellular and molecular levels (Glass et al., 2010). From a broader perspective, a significant amount of evidence suggests that some age-related neurodegenerative diseases, such as PD, are not only CNS disorders but also have systemic pathology and manifestations (Fasano et al., 2015), implying a close link between the non-neuronal periphery and the nervous system during aging. Several studies have highlighted the key roles of AMPs in mediating the upstream signals from non-neuronal tissues/cells to initiate neuronal aging. In *Drosophila*, reflected by a dramatic age-dependent increase of multiple AMPs in the brain, a conserved Relish/NF-κB immune signaling pathway appears to be dysregulated in glial cells during functional aging, and overactivation of the pathway can result in reduced lifespan (Kounatidis et al., 2017). This dysregulation also seems largely responsible for aging-associated neuropathology, as glial-specific overexpression of individual AMP genes known to be regulated by the NF-κB pathway is sufficient to cause the neurodegeneration phenotypes seen in physiological aging (Cao et al., 2013; Kounatidis et al., 2017). More strikingly, our previous studies have discovered an unexpected function of the skin-exclusive AMP, NLP-29, in causatively triggering the aging-associated degeneration of sensory neurons in *C. elegans*, without affecting the lifespan (E et al., 2018) (Table 1). This process requires a neuronal receptor (neuropeptide receptor 12, NPR-12) specific to NLP-29, enabling the transmission of neurodegeneration-initiating signals from the non-neuronal tissues to neurons, via a ligand-receptor binding mechanism (Figure 1). While we find that autophagic machinery is activated downstream of the neuropeptide receptor NPR-12 to mediate neurodegeneration [See (Wauson et al., 2014) for a review of GPCRs in the regulation of autophagy] the role of autophagy in neurodegeneration has been a controversial topic (Wong and Cuervo, 2010) and remains to be comprehensively elucidated in both physiological aging and disease conditions.

Although NLP-29 expression appears to be regulated by a conserved mitogen-activated protein kinase (MAPK) immune signaling pathway in response to pathogenic infections (Pujol et al., 2008), it is unclear whether this pathway also contributes to the age-dependent increase of this “neurodegeneration-causing AMP” in wild-type *C. elegans* (E et al., 2018). One possible source of internal signals that regulate such AMP increases that have regulatory effects on the nervous system, are the hallmarks of aging. These include but are not limited to increased reactive oxygen species, endoplasmic reticulum stress triggered by misfolded protein accumulation, and cellular senescence (López-Otí?n et al., 2013; Childs et al., 2015), which may also induce additional tissue/organ-specific changes on top of the burden generated by aging itself. While it remains to be further investigated whether AMPs contribute to aging-associated neurodegeneration in mammals, our current data suggest that this mechanism may be evolutionarily conserved, as ectopic expression of the *C. elegans* NPR-12/GPCR, in the presence of the ligand NLP-29, stimulates rat cortical neurons to degenerate *in vitro* (E et al., 2018). Considering the conservation of aging-associated expression patterns of AMPs in other organisms, we stress the necessity and importance of future research to explore the crosstalk between AMPs and nervous system in mammalian systems within the context of physiological aging, which likely will add significantly to our understanding of the biological root of neuronal aging.

## 5. Perspectives

In the many years since the discovery of AMPs, researchers have continued to unveil novel functions of these pleotropic molecules. The involvement of AMPs in physiological and pathological aging appears to be a common theme across multicellular organisms, and of recent note, AMPs have garnered more interest in their roles as regulators of the nervous system. Previous studies have highlighted a possibility that AMPs may serve as important signaling molecules that originate from non-neuronal tissues to mediate the progression of aging, neurodegeneration (Figure 1), and subsequent changes in organismal behaviors, while most of these studies investigate such interactions in non-mammalian model systems. With age-dependent increase of various AMPs being a conserved phenomenon across different species in different tissues, as well as the recent evidence implying antimicrobial-related function of the characteristic proteins associated with neurodegenerative diseases, there remain essential and critical questions to be answered from a translational perspective. For instance, what are the evolutionary reasons for AMPs to increase their expression levels in functional aging? What molecular mechanisms regulate AMP expression in the absence of pathogenic infections? Are there any common features among the AMPs that can potentially affect neuronal health in aging, such as requiring specific groups of neuronal surface receptors to mediate the effects? Revealing the mechanisms underlying these intricate phenomena will likely lead to future identification of key biomarkers for preventative health screenings and diagnostics for age-related neurological disorders, and aid in the development of novel therapeutic targets.

## Author Contributions

BS and LE performed the literature search and drafted the manuscript. AF and LE edited the manuscript. All authors contributed to the article and approved the submitted version.

## Funding

This work was supported by the Imagine More Award, Research Affairs Committee, as well as the Department of Cell Biology, Neurobiology, and Anatomy at the Medical College of Wisconsin, and 5520482 Advancing a Healthier Wisconsin (AHW) Endowment project titled Developing Innovative Translational Research Programs in Clinically Relevant Neurological Disorders.

## Conflict of Interest

The authors declare that the research was conducted in the absence of any commercial or financial relationships that could be construed as a potential conflict of interest.

## Publisher's Note

All claims expressed in this article are solely those of the authors and do not necessarily represent those of their affiliated organizations, or those of the publisher, the editors and the reviewers. Any product that may be evaluated in this article, or claim that may be made by its manufacturer, is not guaranteed or endorsed by the publisher.
